# Psychological decoupling in responses to AI: emotional reactivity and behavioral discourse in digital environments

**DOI:** 10.3389/fpsyt.2026.1839044

**Published:** 2026-06-24

**Authors:** Naciye Güliz Ugur, Ismail Bayram

**Affiliations:** Department of Management Information Systems, Sakarya University, Sakarya, Türkiye

**Keywords:** artificial intelligence perception, digital mental health, emotional expression, expressive decoupling, human–AI interaction, sentiment analysis, social media discourse, user-generated discourse

## Abstract

**Introduction:**

Artificial intelligence (AI) has become increasingly visible in digitally mediated environments, raising important questions about how users express emotional reactions to perceived AI presence. While prior research has frequently examined AI acceptance, trust, and behavioral outcomes, less is known about how different forms of AI perception are reflected in large-scale user-generated discourse. This study investigates the relationship between multidimensional AI perception, emotional expression, trust-related discourse, and purchase-related discourse in naturalistic online environments.

**Methods:**

A large-scale dataset consisting of 2,326,781 cleaned YouTube comments collected from 8,947 videos and 5,176 channels was analyzed. AI perception was operationalized through six categories: automation perception, robotic communication style, scriptedness, lack of authenticity, perceived artificiality, and uncanny valley perception. The analytical pipeline combined LLM-assisted weak supervision, transformer-based classification, manual validation, sentiment analysis, and statistical association tests to examine emotional expression and discourse patterns across AI perception categories.

**Results:**

AI perception was highly prevalent throughout the dataset and was strongly associated with negative emotional expression. Comments containing AI perception cues exhibited substantially higher levels of negative sentiment than comments without such cues. Emotional expression varied meaningfully across AI perception categories, with robotic communication style, lack of authenticity, automation perception, and uncanny valley perception showing the highest levels of negative expression. In contrast, trust-related discourse and purchase-related discourse remained rare across the corpus and displayed only limited differentiation across AI perception conditions. These findings indicate that emotional expression is considerably more visible and differentiated than explicit behavioral discourse in AI-related user comments.

**Discussion:**

The findings support a pattern of expressive decoupling, whereby perceived AI presence is strongly reflected in emotional expression while explicit trust-related and purchase-related discourse remains sparse. Rather than indicating actual behavioral outcomes, the results suggest that AI-related perceptions primarily manifest as discursive emotional reactions within AI-saturated digital environments. The study contributes to digital mental health and human–AI interaction research by demonstrating how large-scale user-generated discourse can reveal psychologically meaningful patterns of emotional expression associated with perceived AI presence.

## 1 Introduction

Artificial intelligence (AI) has become an increasingly visible and pervasive component of everyday digital environments, fundamentally reshaping how individuals encounter, interpret, and respond to mediated content (1, 2). As AI-generated and AI-assisted outputs proliferate across social media platforms, recommendation systems, and communication interfaces, users are no longer merely interacting with information or brands; they are increasingly reacting to the perceived presence of non-human agency embedded within these interactions. This shift has important implications for understanding how individuals cognitively and emotionally process technology-mediated experiences, particularly within the emerging domain of digital mental health.

From a psychological perspective, the growing visibility of AI reflects a transition from object-based to agent-based evaluation, in which technological systems are interpreted as quasi-social actors rather than neutral tools (3–6). This transformation has been associated with a range of affective responses, including discomfort, skepticism, and reduced perceived authenticity, often discussed within the broader frameworks of algorithm aversion and the uncanny valley (7–10).

These responses are particularly salient in digitally immersive environments, where emotional reactions are not only internally experienced but also externally expressed through language, interaction, and participation. Recent work in digital mental health further suggests that technology-mediated environments have been associated with emotional regulation patterns and psychological well-being, especially when users are repeatedly exposed to AI-generated or AI-mediated content (11, 12).

Despite this growing body of research, important limitations remain. Much of the existing literature conceptualizes AI perception as a relatively uniform construct, typically operationalized through single dimensions such as perceived artificiality or general AI anxiety. User experiences in real-world digital environments, however, suggest that AI perception is inherently multidimensional, encompassing diverse cues such as robotic communication style, scriptedness, lack of authenticity, perceived artificiality, and uncanny valley responses (6, 13, 14). These perceptual cues may activate distinct cognitive and affective processes, leading to heterogeneous patterns of emotional expression that are not adequately captured by monolithic measures.

A further challenge concerns the assumed continuity between affective responses and downstream behavioral outcomes. Negative emotional reactions toward AI are often expected to translate into reduced trust, avoidance, or diminished engagement. Yet this assumption has rarely been examined using large-scale, naturalistic data and may not hold in digitally saturated environments characterized by low behavioral commitment and high expressive flexibility (15–17). In such contexts, users may articulate strong emotional reactions without corresponding changes in their expressed behavioral orientations.

Importantly, much of the available evidence relies on self-reported intentions or experimental designs, leaving open the question of how these processes unfold in naturally occurring digital discourse. Addressing this gap requires a shift in focus from observed behavior to expressed behavioral discourse—that is, the linguistic articulation of evaluation, intention, and hesitation embedded within user-generated content. Rather than capturing actual behavioral outcomes such as purchasing or disengagement, this approach enables the examination of how individuals express and negotiate their responses to AI within real-world communication environments.

As emphasized in recent methodological and conceptual discussions, user-generated text provides a valuable window into cognitive–affective processes, particularly in large-scale digital contexts where psychological states are externalized through language (18–21).

Building on these perspectives, the present study investigates how the perceived presence of AI is associated with patterns of emotional expression and behavioral discourse in large-scale, naturalistic digital environments. Drawing on a dataset of over 2.3 million user-generated comments, the study develops a multidimensional taxonomy of AI perception and examines how different perception types relate to sentiment and to low-frequency indicators of expressed behavioral orientation, such as purchase-related and trust-related discourse. Importantly, these indicators are conceptualized as discursive proxies, not as direct measures of behavior, and are interpreted accordingly.

The study focuses specifically on AI-saturated digital contexts in which the perception of AI is highly salient. As such, the findings are intended to illuminate psychological response patterns under conditions of sustained AI exposure rather than to generalize across all digital environments. Within this scope, the analysis identifies a consistent pattern: the perception of AI is strongly associated with heightened negative emotional expression (22, 39), yet shows limited and contextually constrained associations with behavioral discourse indicators. This pattern is conceptualized as an expressive decoupling between affective responses and their discursive behavioral articulation (16, 23).

By reframing the relationship between emotion and behavioral expression as a discursive and context-dependent process, this study contributes to research on digital mental health and human–AI interaction in several ways. It advances understanding of how perceived AI presence is associated with emotional expression in naturalistic digital environments, extending the literature beyond controlled experimental settings. It also highlights the importance of distinguishing between internal psychological responses and their external expression in digitally mediated contexts. In addition, the study provides a large-scale empirical basis for examining how cognitive–affective processes unfold in environments characterized by continuous AI exposure and high expressive affordances.

## 2 Literature review and hypothesis development

### 2.1 AI perception in digitally saturated environments

Artificial intelligence (AI) has become increasingly embedded in everyday digital environments, shaping how individuals encounter information, evaluate mediated content, and interpret the agency behind digital interactions (1, 2). Unlike earlier forms of digital technology that often operated as invisible infrastructure, contemporary AI systems are increasingly perceptible to users through linguistic style, automation cues, recommendation patterns, and synthetic or semi-synthetic forms of communication (24).

This visibility has important psychological implications because users do not merely process the content they encounter; they also evaluate the perceived source, intentionality, and authenticity of that content. Research on human–AI interaction indicates that individuals often respond to AI systems through social and psychological categories typically applied to human agents, including trust, competence, warmth, authenticity, and perceived intentionality (3, 13, 14). When AI involvement becomes salient, users may shift from fluent content processing to more critical appraisal, evaluating whether the interaction feels human, authentic, scripted, mechanical, or artificial (6).

Such appraisals are particularly relevant in social media environments, where users routinely express affective reactions, skepticism, humor, discomfort, or resistance through language. Prior research has shown that people may display aversion toward algorithmic systems, especially after perceiving errors or limitations in algorithmic judgment (7, 10). Similarly, studies on medical AI demonstrate that individuals may resist AI involvement in domains perceived to require human sensitivity or individualized understanding (9). Relatedly, the uncanny valley literature suggests that entities perceived as almost human but not fully human can produce discomfort or unease (8). Together, these streams suggest that perceived AI involvement may act as an affective trigger in digitally mediated contexts.

Much of this literature, however, has relied on experimental or self-report designs (15, 16). Although such approaches provide valuable insights and greater control over causal mechanisms, they may not fully capture how AI-related perceptions are expressed in naturally occurring digital discourse. In large-scale online environments, psychological responses are frequently externalized through comments, evaluations, and emotional language (18).

Social media language has therefore become an important source for studying psychological expression at scale, including emotional states, loneliness, well-being, and other mental health-related phenomena (11, 20, 21). This makes user-generated discourse particularly relevant for examining how people express psychological responses to AI-saturated environments.

### 2.2 From monolithic AI perception to multidimensional AI appraisal

A central limitation in existing AI perception research is the tendency to treat AI perception as a single, uniform construct. In many studies, AI perception is captured through broad indicators such as perceived artificiality, algorithm aversion, or general acceptance of AI (7, 10). While useful, this approach may obscure important variation in how users actually experience AI in naturalistic digital settings.

In real-world discourse, users may infer AI presence through multiple perceptual cues. Some may focus on automation, identifying content as mechanically produced or algorithmically generated. Others may react to robotic communication style, repetitive phrasing, scriptedness, lack of authenticity, perceived artificiality, or uncanny human-likeness. These dimensions are psychologically distinct (6, 13). For example, scriptedness may be interpreted as predictable or efficient in some contexts, whereas lack of authenticity may be interpreted as deceptive, emotionally hollow, or manipulative (14). Similarly, robotic communication may signal technical efficiency but may also reduce perceived warmth or relational presence (13).

This distinction is important because it shifts the analytical focus from outcome-oriented interpretations toward a more psychologically grounded account of AI appraisal (6). Rather than examining whether AI perception directly translates into behavioral outcomes, this perspective emphasizes how different forms of AI perception are associated with distinct patterns of emotional expression (13). Such a framing allows for a more precise interpretation of user-generated discourse, recognizing that textual expressions primarily reflect cognitive–affective responses rather than observable behavior (18).

Accordingly, the present study conceptualizes AI perception as a multidimensional appraisal field composed of automation perception, scriptedness, lack of authenticity, robotic communication style, perceived artificiality, and uncanny valley perception. This approach allows the analysis to examine not only whether AI perception is associated with negative emotional expression, but also whether different forms of perceived AI presence are associated with distinct affective patterns.

### 2.3 AI perception and negative emotional expression

Affective responses to AI are often shaped by perceived agency, humanness, authenticity, and control (6, 13, 25). When users detect that content or interaction may be AI-generated, they may experience discomfort, skepticism, reduced trust, or emotional distance. These reactions are consistent with algorithm aversion research, which shows that individuals may lose confidence in algorithmic systems after observing errors, even when those systems perform well overall (7, 10). They are also consistent with research on resistance to medical AI, where users often perceive AI as less capable of recognizing their uniqueness or personal circumstances (9).

In digital environments, these affective responses are not always measured through traditional survey scales. Instead, they may be expressed through language (18). Comments, reactions, and evaluative statements can serve as discursive traces of emotional appraisal (19). Although such expressions should not be equated with clinical states or directly observed behavior, they provide a valuable window into how users articulate emotional responses in naturalistic settings (20, 21).

Within AI-saturated environments, where AI cues are highly salient, users may be especially likely to express negative sentiment when they perceive content as artificial, automated, inauthentic, or robotically produced. Therefore, the first hypothesis focuses specifically on emotional expression rather than behavioral change:

H1. AI perception is positively associated with negative emotional expression in user-generated comments.

### 2.4 Heterogeneity across AI perception types

Although AI perception is often associated with negative affect, not all AI-related cues are likely to produce the same emotional response. Different forms of AI perception may activate different psychological interpretations (6, 13). For instance, perceived artificiality and lack of authenticity may be experienced as violations of sincerity and human presence, thereby increasing negative emotional expression (26, 37). Robotic communication style may similarly reduce perceived warmth and increase discomfort (13). By contrast, scriptedness may sometimes be interpreted as structure, clarity, or predictability, particularly in digital environments where users are accustomed to formulaic content.

This distinction is important because a monolithic treatment of AI perception may mask meaningful heterogeneity (13). If all AI-related perceptions are collapsed into a single category, the analysis may overlook the possibility that some AI cues are emotionally aversive while others are neutral or even positively evaluated.

The present study therefore examines whether sentiment differs across specific AI perception categories.

H2. The association between AI perception and emotional expression varies significantly across different AI perception types.

### 2.5 Behavioral discourse as expressed orientation

A key conceptual issue in this study concerns the interpretation of purchase-related and trust-related expressions. The analysis is based on user-generated textual data and does not capture actual behaviors such as purchasing, disengagement, avoidance, clicks, or conversions. Instead, it examines linguistic expressions embedded in comments (18, 20). Accordingly, purchase-related and trust-related variables are conceptualized as behavioral discourse, referring to expressed orientations, evaluations, or hesitations rather than observed actions (19).

This distinction is particularly important in digitally mediated environments, where users may express emotional reactions without explicitly articulating corresponding behavioral intentions (15, 16). For instance, individuals may display negative affect while continuing to engage with content, or conversely, may not verbalize behavioral considerations despite forming internal evaluations. Such patterns highlight that emotional expression and behavioral discourse represent analytically distinct dimensions of user response (17).

The empirical distribution of these variables also requires careful interpretation. Purchase-related and trust-related discourse appear at relatively low frequencies within the dataset, which limits their suitability for strong inferential claims (17). Therefore, these variables are interpreted as sparse discursive indicators, and the analysis focuses on descriptive and associational patterns rather than causal or predictive relationships.

Accordingly, behavioral discourse is not treated as a mediator or as direct evidence of behavioral outcomes. Instead, it is examined as a secondary layer of expression, allowing the analysis to explore whether affective responses to AI are accompanied by explicit trust-related or purchase-related language in naturally occurring digital discourse (18, 19).

### 2.6 AI perception and trust-related discourse

Trust is central to human–AI interaction because users must evaluate whether AI systems are competent, reliable, transparent, and aligned with human expectations (3, 27). Prior research suggests that trust in AI depends on system representation, task type, perceived intelligence, reliability, and the degree to which users believe the system can perform appropriately in a given context (3). In sensitive or relational domains, AI involvement may reduce trust because users may perceive AI as lacking empathy, contextual understanding, or human judgment.

In naturalistic digital discourse, however, trust is not always expressed directly (18). Users may not explicitly state that they trust or distrust a system. Instead, trust-related discourse may appear through comments questioning authenticity, credibility, manipulation, deception, or reliability (19). Because such signals are rare and context-dependent, they should be interpreted cautiously (17).

Rather than proposing a strong directional behavioral hypothesis, the present study formulates this issue as a research question:

RQ1. How is AI perception associated with trust-related discourse in user-generated comments?

This formulation avoids overstating the behavioral meaning of rare textual signals while still allowing the study to examine whether trust-related expressions appear alongside AI perception (17, 18).

### 2.7 AI perception and purchase-related discourse

Purchase-related discourse represents another form of expressed behavioral orientation. In online comments, users may occasionally express willingness to buy, reluctance to purchase, avoidance, recommendation, or hesitation (15, 28). However, such expressions should not be treated as actual purchasing behavior. They are linguistic indicators of possible behavioral orientation, not observable action (16, 17).

This distinction is especially important in AI-saturated social media environments. Users may react emotionally to AI-generated or AI-assisted content without translating those reactions into explicit purchase-related language (19, 23). Social media platforms are often low-commitment expressive spaces where users can criticize, joke, or express discomfort without making concrete decisions (29). Emotional expression may therefore function more as social interaction than as a direct precursor to economic action (19).

Given the very low frequency of purchase-related discourse in the dataset, this variable requires careful and restrained interpretation. Accordingly, the analysis approaches purchase-related expressions as sparse discursive indicators and focuses on descriptive and associational patterns rather than indirect or causal relationships (17).

Within this framework, the study examines whether purchase-related language co-occurs with AI perception in naturally occurring user-generated discourse (18).

RQ2. How is AI perception associated with purchase-related discourse in user-generated comments?

### 2.8 Expressive decoupling between emotional expression and behavioral discourse

The central theoretical contribution of this study lies in identifying a potential form of expressive decoupling between emotional expression and behavioral discourse in AI-saturated digital environments. In this context, expressive decoupling refers to a pattern in which AI perception is strongly associated with negative emotional language while showing comparatively weak or inconsistent associations with explicit trust-related or purchase-related discourse (16, 17).

This perspective aligns with research on digital psychological expression, where user-generated language is understood as an externalization of cognitive–affective appraisal rather than a direct reflection of observable behavior (18, 20). Within digitally mediated environments, emotional reactions may become highly visible in discourse even when corresponding behavioral orientations remain implicit, ambiguous, or unarticulated (15).

Several contextual dynamics may contribute to this pattern. Users may express affective discomfort while simultaneously treating AI-generated or AI-assisted content as routine, entertaining, or unavoidable within platform environments. In addition, social media spaces often facilitate rapid emotional expression without requiring concrete behavioral commitment (19, 23). Continued exposure to AI-mediated communication may also contribute to the coexistence of negative affect and ongoing participation within digital environments.

These interpretations are conceptual rather than causal. Accordingly, the present study does not attempt to infer actual behavioral outcomes. Instead, it examines whether the observed distribution of emotional expression and behavioral discourse is consistent with the notion of expressive decoupling in large-scale user-generated discourse.

H3. AI perception shows a stronger and more consistent association with negative emotional expression than with trust-related and purchase-related discourse indicators.

### 2.9 Descriptive temporal patterns in AI-related emotional expression

Finally, the study examines temporal patterns in AI-related discourse. The temporal analysis is approached descriptively and is intended to identify observable variation in emotional expression and AI-related comment volume across different periods within the dataset (30).

Because the analysis does not control for contextual factors such as platform policy changes, algorithmic shifts, changes in video content, or broader societal developments, the findings are not interpreted causally. Rather than inferring directional effects, the temporal perspective provides contextual insight into how AI-related discourse may fluctuate over time within digitally saturated environments (30).

Examining these descriptive patterns is particularly relevant given the increasing visibility of AI technologies in everyday digital communication and the evolving ways users respond to AI-mediated content.

RQ3. How do AI-related emotional expressions and comment volumes vary descriptively over time?

## 3 Methods

### 3.1 Research design

This study adopts a large-scale observational research design to examine how perceived AI presence is reflected in naturalistic digital discourse and how it relates to emotional expression and behavioral discourse. Rather than measuring observable behavioral outcomes such as purchasing, disengagement, clicks, or conversions, the study analyzes user-generated textual expressions as discursive indicators of psychological appraisal, emotional response, trust-related concern, and purchase-related orientation (18, 20).

The research design follows a computational social science approach integrating weak supervision, large language model-assisted pre-labeling, transformer-based classification, manual validation, and descriptive-comparative statistical analysis (31–34, 38). This approach is particularly suitable for investigating psychological expression in large-scale digital environments because AI-related perceptions frequently emerge spontaneously within user discourse rather than through researcher-directed self-report instruments (18). By analyzing organically occurring comments, the study captures naturally expressed affective and evaluative reactions within an AI-saturated platform context.

All statistical analyses are interpreted as associational rather than causal. The observational nature of the dataset, the platform-specific sampling structure, and the reliance on textual indicators limit causal inference (31). Accordingly, the analysis focuses on identifying patterns of association among AI perception categories, emotional expression, and low-frequency behavioral discourse indicators.

This analytical perspective reflects the distinction between expressed psychological orientation and observable behavior in digitally mediated communication environments (17).

### 3.2 Data collection

The empirical context of the study consists of user comments collected from highly viewed YouTube video content. YouTube was selected because it provides a large-scale, publicly accessible environment in which users spontaneously express reactions to digital content, platform-mediated communication, and perceived artificiality (1). The platform is particularly suitable for examining AI perception because users frequently articulate judgments about authenticity, automation, scriptedness, and artificiality in comment sections (24).

Data were collected through the official YouTube Data API in accordance with platform access rules. The initial dataset included 3,517,642 comments posted under 8,947 videos across 5,176 channels. The videos were identified using 47 search criteria related to the substantive domain of the study. These search criteria were used to capture a broad set of videos with substantial audience reach and sufficient user interaction. The use of highly viewed videos increased the likelihood of obtaining diverse, unsolicited, and naturally occurring user responses (20).

The raw dataset included comment text, video-level identifiers, channel-level identifiers, comment-level visibility indicators, and available metadata required for preprocessing and analysis. No private, password-protected, or non-public user data were collected.

The unit of analysis is the individual user comment. The study analyzes textual content at scale and does not attempt to identify individual users (18).

The complete list of YouTube search criteria and additional technical details regarding the data collection pipeline are provided in Appendices A and B of the **Supplementary Material**. Reproducibility materials, analytical code, annotation resources, and supplementary documentation are publicly available through the Zenodo repository (https://doi.org/10.5281/zenodo.20424036).

### 3.3 Inclusion, exclusion, and data cleaning criteria

The raw corpus was cleaned through a multi-stage preprocessing pipeline designed to improve linguistic quality while preserving psychologically meaningful expressions (18). Comments were excluded if they met one or more of the following criteria: non-English language, duplicate or near-duplicate content, bot-like or spam-like entries, extremely low-information text, or comments consisting primarily of URLs, symbols, or non-linguistic artifacts.

The preprocessing procedure included language filtering, deduplication, normalization, tokenization, removal of URLs and non-textual artifacts, and standard text cleaning. However, the preprocessing strategy was intentionally conservative. Terms and expressions relevant to AI perception, such as “AI,” “robotic,” “fake,” “scripted,” “automated,” “artificial,” and similar cues, were retained. This was necessary because overly aggressive cleaning could remove precisely the linguistic markers needed to detect perceived artificiality and related psychological appraisals (20).

After preprocessing, the cleaned dataset consisted of 2,326,781 comments associated with 8,417 videos and 4,853 channels. The cleaned corpus retained the full set of 47 search criteria and included 1,786,342 unique commenters. Thus, data cleaning improved textual quality without substantially narrowing topical coverage or eliminating the large-scale nature of the dataset.

### 3.4 Operationalization of AI perception

#### 3.4.1 Multidimensional taxonomy development

AI perception was operationalized as a multidimensional discursive construct rather than as a single binary or monolithic indicator (6, 13). Based on the theoretical framing of AI perception as a heterogeneous appraisal field, six categories were defined:

- automation perception
- robotic communication style
- scriptedness
- lack of authenticity
- perceived artificiality
- uncanny valley perception

These categories reflect different ways in which users linguistically express perceived AI presence (18).

Automation perception captures comments referring to mechanized or algorithmic production. Robotic communication style captures comments describing communication as mechanical, unnatural, or non-human. Scriptedness captures perceptions of formulaic, repetitive, or pre-planned communication. Lack of authenticity captures comments suggesting insincerity, falseness, or the absence of genuine human presence. Perceived artificiality captures explicit recognition of artificial or synthetic qualities. Uncanny valley perception captures discomfort arising from entities or content perceived as nearly human but not fully human.

The taxonomy was developed through an iterative process combining theory-driven category specification, manual inspection of comments, keyword seeding, weak supervision rules, and transformer-based classification refinement (31). This approach allowed the study to retain theoretical coherence while remaining sensitive to the language users actually employ in naturalistic digital discourse.

Operational definitions and illustrative examples for each AI-perception category are provided in Appendix D of the **Supplementary Material**.

#### 3.4.2 Weak supervision and LLM-assisted pre-labeling

Because fully manual annotation of more than 2.3 million comments was not feasible, the study employed a weak supervision strategy (32). An initial pre-labeling phase was conducted using Ollama (version 0.9.3) running Gemma 3:4B-Instruct. This model was used to generate preliminary binary labels for each AI perception category. The pre-labels were not treated as ground truth; rather, they served as weak supervision signals for scalable classification (32).

For each category, labeling instructions were designed to identify whether a comment contained linguistic evidence corresponding to the relevant AI perception dimension. The model was instructed to assign binary labels indicating the presence or absence of each category. The prompt logic focused on semantic meaning rather than keyword matching alone, allowing the pre-labeling stage to capture implicit expressions of artificiality, automation, or authenticity concerns (18).

This stage was necessary because AI perception signals are often context-dependent and may be expressed through subtle wording. However, because LLM-generated labels may contain noise, the pre-labeling outputs were subjected to manual validation before downstream modeling.

The full weak supervision prompt structure and LLM-assisted pre-labeling procedure are documented in Appendix E of the **Supplementary Material**.

#### 3.4.3 Manual validation procedure

To evaluate the reliability of the LLM-assisted pre-labeling process, a structured manual validation procedure was implemented. For each AI perception category, 250 positive and 250 negative instances were randomly sampled from the pre-labeled dataset. These instances were independently reviewed by human coders using the category definitions described above. The purpose of this validation stage was to assess whether the machine-generated labels aligned with human interpretation.

Model-generated labels were compared with manual judgments using standard classification metrics, including accuracy, precision, recall, and F1-score (33). The validation results indicated that the pre-labeling process achieved acceptable performance across categories, with accuracy and F1-scores generally ranging between 86% and 91%. This validation procedure supports the use of weak supervision as a scalable method for identifying complex AI perception signals in large-scale discourse (32).

At the same time, the study acknowledges that AI perception categories may overlap semantically and that some boundary cases may remain difficult to classify with complete certainty. The inter-rater agreement between human coders, calculated using Cohen’s Kappa, was κ = .76, indicating substantial agreement.

Additional details regarding the manual validation procedure and inter-rater agreement statistics are reported in Appendix F of the **Supplementary Material**.

#### 3.4.4 Supervised classification using RoBERTa

Following pre-labeling and manual validation, a RoBERTa-base model was fine-tuned to classify comments into AI perception categories. Model training and evaluation were implemented in Python (version 3.7), using PyTorch (version 2.6.0) and Hugging Face Transformers (version 4.51.3). RoBERTa was selected because transformer-based models are well suited for capturing contextual meaning in short, noisy, and semantically variable social media text (33).

The model was trained using a maximum sequence length of 128 tokens. To address class imbalance and reduce overfitting, dropout of 30%, class weighting, and label smoothing with ϵ = 0.1 were applied. The model was optimized using AdamW with a learning rate of 2e−5 over four training epochs. A stratified data split was used, with 20% of the data reserved for testing and 10% of the remaining training data used for validation. Early stopping was applied when validation performance failed to improve across two consecutive evaluation steps.

Model performance was evaluated using accuracy, precision, recall, F1-score, and confusion matrix inspection (33). Given the class imbalance across some categories, F1-score and recall were prioritized over accuracy alone.

Qualitative error inspection was also conducted to identify common boundary cases. Most classification ambiguity occurred when comments contained multiple overlapping AI perception cues, such as both artificiality and lack of authenticity.

Detailed transformer training settings, evaluation metrics, and classification-threshold procedures are reported in Appendix G of the **Supplementary Material**.

### 3.5 Sentiment measurement

Emotional expression was measured using a transformer-based sentiment classification model calibrated for social media language (33). Sentiment was operationalized as a binary indicator distinguishing negative from positive emotional expression. The decision to focus on negative emotional expression reflects the theoretical aim of the study: to examine whether perceived AI presence is associated with adverse affective appraisal in user-generated discourse.

This measure should be interpreted as emotional expression in text rather than as a clinical or diagnostic indicator of mental health status (18, 20). The study does not infer psychiatric symptoms, diagnoses, or individual-level psychological conditions. Instead, sentiment classification is used to capture large-scale patterns of affective language in an AI-saturated digital environment.

Additional details regarding the sentiment-analysis pipeline are provided in Appendix H of the **Supplementary Material**.

### 3.6 Behavioral discourse signal construction

The study constructed two additional textual indicators: trust-related discourse and purchase-related discourse. These indicators were included to examine whether negative emotional expression is accompanied by explicit behavioral orientation within user-generated comments. However, these indicators are not interpreted as direct measures of observable behavior (16, 17).

Instead, they are conceptualized as forms of behavioral discourse, reflecting textual expressions of trust-related concern, purchase-related intention, hesitation, or evaluative orientation (18, 19). This distinction is methodologically important because the dataset does not include observable actions such as transactions, purchasing behavior, clicks, conversions, avoidance, or platform disengagement.

Consequently, the analysis focuses on discursive patterns embedded in language rather than on actual behavioral outcomes.

#### 3.6.1 Trust-related discourse

Trust-related discourse was identified using a hybrid rule- and context-based classification procedure. The dictionary component captured explicit linguistic markers of distrust, credibility concern, manipulation, deception, or skepticism (27). The contextual component was used to distinguish genuine trust-related expressions from ambiguous mentions.

This variable captures whether a comment contains an explicit trust-related concern. It does not measure actual trust as a psychological state, nor does it measure behavioral withdrawal from a brand, platform, or AI system (17).

Because trust-related discourse is rare in the dataset, all analyses involving this variable are interpreted cautiously and with attention to absolute frequencies and practical significance.

Rule-based trust-related discourse detection procedures are documented in Appendix I of the **Supplementary Material**.

#### 3.6.2 Purchase-related discourse

Purchase-related discourse was operationalized as explicit textual reference to buying intention, purchase consideration, purchase avoidance, recommendation, or acquisition-related evaluation (15, 28). A combination of keyword expansion and contextual filtering was used to distinguish genuine purchase-related expressions from metaphorical or irrelevant uses of purchase-related language.

This measure captures expressed purchase orientation, not actual purchase behavior (16). Because purchase-related discourse occurs at a very low base rate in the dataset, it is treated as a sparse discursive indicator (17).

Accordingly, the analysis does not use this variable to infer actual behavioral outcomes or behavioral change.

Additional purchase-related discourse detection rules and contextual filtering procedures are documented in Appendix I of the **Supplementary Material**.

### 3.7 Contextual and comment-level descriptors

Several comment-level and context-level descriptors were retained to characterize the corpus and to support descriptive interpretation, including comment length, temporal indicators, and video-level clustering information. Comment length was included because longer comments may contain more explicit evaluative language and may therefore be more likely to include AI perception or behavioral discourse signals (18). Comment-level visibility indicators were retained only as contextual descriptors and were not interpreted as behavioral outcomes.

Temporal indicators were included to capture changes in discourse over time. Video-level clustering adjustments were used because comments under the same video may share contextual features.

### 3.8 Analytical strategy

The analytical procedure was designed to characterize the dataset, examine patterns of emotional expression, and evaluate the distribution of behavioral discourse indicators. Descriptive statistics were used to summarize the structure of both the raw and cleaned datasets, including comment counts, video counts, channel counts, user counts, and comment-level visibility indicators. AI perception categories and sentiment distributions were then examined to identify patterns of emotional expression across perception types.

Trust-related and purchase-related discourse were subsequently analyzed as low-frequency textual indicators. In addition, descriptive temporal analyses were conducted to explore variation in AI-related emotional expression and comment volume over time.

For categorical associations, chi-square tests were used together with distributional comparisons. However, given the very large sample size, statistical significance alone was not treated as sufficient evidence of substantive importance (31). Accordingly, the analysis emphasizes effect sizes, absolute percentage differences, absolute risk differences, and relative risk ratios where appropriate. This approach reduces the likelihood of overinterpreting statistically significant but practically limited differences.

For sparse binary outcomes, particularly trust-related and purchase-related discourse, interpretation focuses primarily on base rates, absolute differences, and descriptive patterns rather than strong inferential claims (17). Because these forms of discourse occur at relatively low frequencies within the corpus, they are interpreted cautiously as sparse discursive indicators rather than robust behavioral signals.

The analytical framework does not examine indirect or mediation-based relationships among variables. Instead, the analysis focuses on descriptive and associational patterns linking AI perception, emotional expression, and behavioral discourse indicators. Particular attention is given to the relative prominence of emotional expression compared with trust-related and purchase-related discourse within the dataset.

All findings are interpreted within the constraints of an observational, platform-specific, and AI-saturated dataset. Accordingly, the study identifies patterns of association in user-generated discourse rather than causal effects of AI exposure.

### 3.9 Robustness and validation checks

Several robustness and validation checks were conducted to assess the stability and credibility of the findings. Classification performance was evaluated using standard metrics, including accuracy, precision, recall, and F1-score (33). The quality of the LLM-assisted pre-labeling process was further assessed through manual validation procedures (32).

Additional category-level analyses were performed to evaluate whether the observed results were disproportionately influenced by any single AI perception category. Descriptive comparisons across categories were also examined to determine whether the central pattern identified in the study—the stronger association between AI perception and negative emotional expression relative to behavioral discourse indicators—remained stable across analytical conditions.

Given the class imbalance present in the dataset, robustness checks were interpreted with particular attention to low base-rate outcomes. For trust-related and purchase-related discourse, the analysis emphasizes that rare textual signals should not be overgeneralized (17).

The purpose of these robustness procedures is not to establish causal or behavioral effects. Rather, they are intended to evaluate the consistency and credibility of the observed discursive patterns.

### 3.10 Ethical considerations

The study used publicly available YouTube comments collected through the official YouTube Data API. No private, password-protected, or personally identifiable information was analyzed, and all analyses were conducted exclusively at the aggregate level without any attempt to identify, profile, evaluate, or diagnose individual users.

Although the data were publicly accessible, the study recognizes that large-scale digital traces may still involve ethical sensitivities related to behavioral inference and user expression. Accordingly, the findings should be interpreted as aggregate-level patterns of textual expression rather than as individual-level psychological or psychiatric assessments (18, 20).

This distinction is particularly important given the journal context. The study contributes to digital mental health and human–AI interaction research by examining emotional expression in digital environments, but it does not make clinical claims regarding users’ mental health status.

To support transparency and reproducibility, detailed methodological documentation, analytical code, annotation resources, prompts, and **Supplementary Materials** are provided through the **Supplementary Material** and the publicly accessible Zenodo repository (https://doi.org/10.5281/zenodo.20424036).

## 4 Results

### 4.1 Raw dataset characteristics

**Table 1** presents the descriptive characteristics of the raw dataset collected from YouTube comments. The initial corpus consisted of 3,517,642 comments posted by 2,427,318 unique commenters. These comments were associated with 8,947 videos across 5,176 channels and were retrieved using 47 search criteria. The scale and diversity of the raw dataset indicate broad coverage of user-generated discourse and provide a sufficiently large corpus for identifying both frequent emotional expressions and comparatively low-frequency discourse signals.

**Table 1:** Raw dataset statistics.

Variable	Value
Number Of Different Videos	8,947
Number Of Unique Commenters	2,427,318
Total Number Of Comments	3,517,642
Number Of Different Channels	5,176
Number Of Different Search Criteria	47
Max Comment Length (Words)	2,187
Minimum Comment Length (Words)	1
Average Comment Length (Words)	17
Average Comment Length (Characters)	92

Comment length varied substantially, ranging from 1 to 2,187 words, with an average length of 17 words and 92 characters. This variation suggests that the dataset contains both brief affective reactions and longer evaluative or interpretive statements, both of which are relevant for examining AI-related psychological expression in naturalistic digital environments.

Overall, the raw corpus provides a large-scale and discourse-rich foundation for analyzing emotional expression and behavioral discourse within AI-saturated digital contexts.

### 4.2 Cleaned dataset characteristics

After preprocessing, the cleaned dataset consisted of 2,326,781 comments associated with 8,417 videos and 4,853 channels, as detailed in **Table 2**. The cleaned corpus retained the same 47 search criteria, indicating that preprocessing improved textual quality without substantially narrowing the thematic breadth of the dataset. The number of unique commenters remained high at 1,786,342, supporting the diversity and scale of user-generated discourse within the final analytical sample.

**Table 2:** Cleaned dataset statistics.

Variable	Value
Number Of Different Videos	8,417
Number Of Unique Commenters	1,786,342
Total Number Of Comments	2,326,781
Number Of Different Channels	4,853
Number Of Different Search Criteria	47
Max Comment Length (Words)	1,943
Minimum Comment Length (Words)	1
Average Comment Length (Words)	21
Average Comment Length (Characters)	112

The cleaned dataset preserved substantial linguistic diversity and contextual breadth. Following preprocessing, the average comment length increased from 17 to 21 words and from 92 to 112 characters. This increase suggests that the preprocessing pipeline effectively removed low-information, repetitive, or noisy entries while preserving semantically richer and more linguistically interpretable comments.

Overall, the cleaned dataset provides a large-scale and discourse-rich foundation for examining emotional expression and behavioral discourse in AI-saturated digital environments.

### 4.3 Weak supervision and manual validation of AI-perception labels

The first stage of model development involved LLM-assisted pre-labeling using the Ollama Gemma 3:4B model. This stage generated preliminary binary labels for each AI perception category and served as a weak supervision mechanism rather than a source of final ground-truth labels. The distribution of pre-labeled data indicated substantial variation across AI perception categories, suggesting that different forms of AI perception were not equally represented in the corpus.

To evaluate the quality of the pre-labeling process, a structured manual validation procedure was conducted. For each AI perception category, 250 positive and 250 negative instances were randomly sampled and independently reviewed by human coders. Machine-generated labels were then compared with human judgments using accuracy, precision, recall, and F1-score.

The validation results indicated acceptable performance across the six AI perception categories, with accuracy and F1-scores generally ranging between 86% and 91%. Performance was stronger for more explicit categories and slightly weaker for more abstract or semantically overlapping categories. This pattern is expected given the complexity of AI perception as a multidimensional construct.

Overall, the manual validation results support the use of weak supervision as a scalable strategy for detecting AI perception in large-scale user-generated discourse, while also indicating the need to interpret category-level classifications with appropriate caution.

### 4.4 Supervised classification model training

Following weak supervision and manual validation, a RoBERTa-base model was fine-tuned to classify AI perception categories in the full cleaned corpus. Model performance was assessed using accuracy, precision, recall, F1-score, and confusion matrix inspection. Given the class imbalance across perception categories, F1-score and recall were prioritized over accuracy alone.

The supervised classification results indicated stable and satisfactory performance across the perception categories. Most classification ambiguity occurred in comments where multiple AI perception cues co-occurred, such as artificiality and lack of authenticity, or scriptedness and robotic communication style. These overlaps are conceptually meaningful and reflect the inherently multidimensional nature of AI perception in naturalistic discourse.

The model was therefore considered suitable for large-scale classification, while the subsequent analyses were interpreted as pattern detection in user-generated language rather than as exact measurement of internal psychological states.

### 4.5 Descriptive distribution of core variables

**Table 3** presents the descriptive distribution of the core analytical variables. AI perception was present in 2,283,054 comments, corresponding to 98.12% of the cleaned dataset. Only 43,727 comments, or 1.88%, were classified as not containing AI perception cues. This distribution indicates that the dataset represents an AI-saturated digital context in which references to artificiality, automation, and AI-mediated communication are highly salient within user discourse. Accordingly, the findings should be interpreted as reflecting user expression under conditions of high AI visibility rather than as broadly representative of digital environments where AI-related cues are less prevalent.

**Table 3:** Descriptive distribution of core variables.

Variable	Value	Frequency	Percentage
AI Perception	True	2,283,054	98.12
	False	43,727	1.88
Sentiment	Negative	1,545,112	66.41
	Positive	781,669	33.59
Purchasing Statement	Neutral	2,318,146	99.63
	Positive	7,181	0.31
	Negative	1,454	0.06
Trust-Related Discourse	False	2,309,524	99.26
	True	17,257	0.74
AI Type	Automation_Perception	681,211	29.84
	Scriptedness	483,259	21.17
	Uncanny_Valley_Perception	367,085	16.08
	Perceived_Artificiality	306,093	13.41
	Lack_of_Authenticity	247,077	10.82
	Robotic_Communication_Style	198,329	8.68

Negative emotional expression was also dominant throughout the corpus. A total of 1,545,112 comments, corresponding to 66.41%, were classified as negative, while 781,669 comments, or 33.59%, were classified as positive. This distribution suggests that negative emotional language is considerably more prevalent than positive emotional language within the AI-saturated communicative environment represented by the dataset.

In contrast, behavioral discourse indicators remained highly infrequent. Purchase-related discourse was absent in 99.63% of comments. Positive purchase-related discourse appeared in only 0.31% of comments, while negative purchase-related discourse appeared in just 0.06%. Trust-related discourse was similarly sparse, with trust-related discourse signals identified in only 0.74% of comments. These distributions indicate that explicit behavioral discourse represents only a small fraction of user-generated responses, despite the widespread presence of emotional expression.

The distribution of AI perception categories further demonstrated substantial heterogeneity. Automation perception was the most frequent category, followed by scriptedness, uncanny valley perception, perceived artificiality, lack of authenticity, and robotic communication style. These distributions reinforce the conceptualization of AI perception as a multidimensional appraisal structure rather than a single uniform perceptual construct.

### 4.6 AI perception and negative emotional expression

H1 proposed that AI perception would be positively associated with negative emotional expression in user-generated comments. A chi-square test of independence indicated a statistically significant association between AI perception and sentiment polarity, χ² = 26,652.94, df = 1, p <.001, φ = .11. Although the phi coefficient indicates a modest effect size in conventional statistical terms, the observed percentage differences remain substantively meaningful given the scale and consistency of the emotional-expression contrast across the corpus.

Given the very large sample size, statistical significance alone is not sufficient for interpretation. Therefore, the practical magnitude of the association was evaluated using absolute percentage differences, risk difference, and relative risk estimates.

As shown in **Table 4**, comments containing AI perception exhibited a substantially higher proportion of negative emotional expression than comments without AI perception. Among comments with AI perception, 67.12% were classified as negative, compared with 29.88% among comments without AI perception. This corresponds to an absolute risk difference of 37.24 percentage points. The relative risk ratio indicates that negative emotional expression was approximately 2.25 times more common in comments containing AI perception than in comments without AI perception.

**Table 4:** AI perception and negative emotional expression.

AI_Perception	Negative_N	Negative_%	Positive_N	Positive_%	Total_N
False	13,065	29.88	30,662	70.12	43,727
True	1,532,047	67.12	751,007	32.88	2,283,054
Chi-square	26,652.94				
p-value	<.001				
df	1				
Effect size (Phi):	.11				

Although the phi coefficient remained modest in conventional terms due to class imbalance and the scale of the dataset, the observed difference in emotional expression is substantively meaningful. The magnitude of the percentage difference suggests that AI perception is associated with markedly higher levels of negative emotional expression within the discourse environment examined in this study.

These findings support H1. AI perception is associated with substantially higher levels of negative emotional expression in user-generated comments. Importantly, this finding should be interpreted as an association within textual and discursive expression rather than as evidence that AI-related discourse cues directly or causally generate negative emotional states.

### 4.7 Emotional expression across AI perception types

H2 proposed that the association between AI perception and emotional expression would vary across different AI perception types. To evaluate this hypothesis, sentiment distributions were examined across the six AI perception categories.

**Table 5** demonstrates substantial heterogeneity in emotional expression across AI perception types. Negative emotional expression was highest for robotic communication style, where 78.41% of comments were classified as negative. Lack of authenticity also showed a strongly negative emotional profile at 75.26%, followed by uncanny valley perception at 72.83% and automation perception at 71.94%. These categories appear to be associated with more adverse emotional expression, particularly in contexts where users perceive communication as mechanical, emotionally detached, artificial, or insufficiently human.

**Table 5:** Category-level sentiment distributions.

Category	Negative	Negative %	Negative mean probability	Positive	Positive %	Positive mean probability	Total
Robotic_Communication_Style	155,525	78.41	0.76	42,804	21.59	0.74	198,329
Lack_of_Authenticity	185,940	75.26	0.58	61,137	24.74	0.56	247,077
Automation_Perception	490,183	71.94	0.71	191,028	28.06	0.69	681,211
Uncanny_Valley_Perception	267,340	72.83	0.89	99,745	27.17	0.88	367,085
Perceived_Artificiality	187,506	61.27	0.84	118,587	38.73	0.82	306,093
Scriptedness	203,880	42.18	0.67	279,379	57.82	0.73	483,259

By contrast, perceived artificiality exhibited a comparatively more balanced emotional distribution, with 61.27% negative and 38.73% positive comments. Scriptedness displayed the most distinctive pattern among all AI perception categories: only 42.18% of scriptedness-related comments were classified as negative, while 57.82% were positive. This pattern suggests that structured, repetitive, or formulaic communication is not uniformly experienced as aversive and may, in some contexts, be interpreted as clear, predictable, or functionally acceptable.

Overall, these findings support H2. AI perception is not associated with a single uniform emotional response; rather, emotional expression varies meaningfully across perception types. In percentage terms, negative emotional expression was highest for robotic communication style, followed by lack of authenticity, uncanny valley perception, and automation perception. The comparatively positive profile of scriptedness further reinforces the multidimensional nature of AI perception and suggests that different AI-related cues may evoke qualitatively different forms of emotional appraisal.

### 4.8 AI perception and trust-related discourse

RQ1 examined how AI perception is associated with trust-related discourse in user-generated comments. **Table 6** presents the cross-tabulation between AI perception and trust-related discourse.

**Table 6:** AI perception and trust-related discourse.

AI perception	Trust_Related_Discourse_Present_N	Trust_Related_Discourse_Present_%	Trust_Related_Discourse_Absent_N	Trust_Related_Discourse_Absent_%	Total_N
True	17,025	0.75	2,266,029	99.25	2,283,054
False	232	0.53	43,495	99.47	43,727
Odds_Ratio	1.41				
Chi-square	26.98				
p-value	<.001				
df	1				
Effect size (Phi):	.003				

Overall, trust-related discourse remained highly infrequent throughout the dataset. Among comments containing AI perception, trust-related discourse appeared in 0.74% of comments. Among comments without AI perception, trust-related discourse appeared in 0.53% of comments. The absolute risk difference between the two groups was 0.21 percentage points. The relative risk ratio was approximately 1.40, and the odds ratio was 1.41.

A chi-square test indicated a statistically significant association between AI perception and trust-related discourse, χ² = 26.98, df = 1, p <.001, φ = .003. Despite statistical significance, the effect size remained extremely small, reflecting the very low base rate of trust-related discourse across the corpus.

Accordingly, the answer to RQ1 should be interpreted cautiously. AI perception demonstrates only a weak association with explicit trust-related discourse in user-generated comments. This finding should not be interpreted as evidence of actual trust formation, trust erosion, or behavioral withdrawal. Rather, it suggests that explicit trust-related language remains relatively uncommon in this type of naturally occurring digital discourse, even within highly AI-saturated communicative environments.

### 4.9 Category-level trust-related discourse

To further examine RQ1, trust-related discourse was analyzed across AI perception categories. As shown in **Table 7**, trust-related discourse remained infrequent across all categories, although modest variation was observed.

**Table 7:** Category-level trust-related discourse.

AI_Perception_Type	Trust_Related_Discourse_Present_N	Trust_Related_Discourse_Present_%	Trust_Related_Discourse_Absent_N	Trust_Related_Discourse_Absent_%	Total_N
Perceived_Artificiality	5,940	1.94	300,153	98.06	306,093
Lack_Of_Authenticity	2,601	1.05	244,476	98.95	247,077
Scriptedness	3,518	0.73	479,741	99.27	483,259
Automation_Perception	3,986	0.59	677,225	99.41	681,211
Robotic_Communication_Style	598	0.30	197,731	99.70	198,329
Uncanny_Valley_Perception	382	0.10	366,703	99.90	367,085
Chi-square	9,029.05				
p-value	<.001				
df	5				
Effect size (Cramér’s V):	.06				

Perceived artificiality exhibited the highest rate of trust-related discourse at 1.84%, followed by lack of authenticity at 0.96% and scriptedness at 0.71%. Automation perception showed a comparatively lower rate of 0.58%, while robotic communication style and uncanny valley perception demonstrated the lowest rates at 0.29% and 0.12%, respectively.

Although the chi-square test indicated statistically significant differences across AI perception categories (χ² = 9,029.05, df = 5, p <.001, Cramér’s V = .06), the practical magnitude of the association remained limited. The primary finding is not that AI perception categories produce strong trust-related outcomes, but rather that explicit trust-related language appears somewhat more frequently in categories associated with perceived artificiality and authenticity-related concerns.

Overall, the category-level findings suggest that trust-related discourse is most likely to emerge when users explicitly frame content as artificial, deceptive, or insufficiently authentic. At the same time, the very low absolute percentages observed across all categories indicate that trust-related discourse remains a relatively uncommon form of response within this AI-saturated digital environment. These results therefore reinforce the broader pattern observed throughout the study: emotional expression is substantially more prevalent and differentiated than explicit trust-related discourse.

### 4.10 AI perception and purchase-related discourse

The relationship between AI perception and purchase-related discourse was examined to assess whether emotional reactions are accompanied by explicit behavioral expressions within user-generated comments. As shown in **Table 8**, purchase-related discourse remained overwhelmingly absent across the dataset.

**Table 8:** AI perception and purchase-related discourse.

AI perception	Negative_N	Negative_%	Neutral_N	Neutral_%	Positive_N	Positive_%	Total_N
False	4	0.01	43,709	99.96	14	0.03	43,727
True	1,450	0.06	2,274,437	99.63	7,167	0.31	2,283,054
Chi-square	131.26						
p-value	<.001						
df	2						
Effect size (Cramér’s V):	.008						

Among comments containing AI perception, 99.63% did not contain purchase-related expressions, while positive purchase-related discourse appeared in only 0.31% of comments and negative purchase-related discourse in 0.06%. Among comments without AI perception, purchase-related discourse was nearly absent, with 99.96% of comments classified as neutral and only a very small number containing positive purchase-related language.

Although the chi-square test indicated a statistically significant association between AI perception and purchase-related discourse (χ² = 131.26, df = 2, p <.001, Cramér’s V = .008), the effect size remained negligible in practical terms. Given the extremely large sample size and the highly sparse distribution of purchase-related discourse, statistical significance should not be interpreted as evidence of a substantively meaningful relationship. The observed differences remain limited within a low base-rate discourse environment.

Instead, the primary finding emerging from these results is the structural rarity of explicit purchase-related language within the corpus. While users frequently express emotional reactions to perceived AI-generated or AI-assisted content, they rarely articulate corresponding purchase intentions, purchase hesitation, or explicit behavioral evaluations.

Overall, these findings suggest that AI perception operates more strongly at the level of affective appraisal than at the level of explicit behavioral discourse. Emotional responses to perceived artificiality therefore appear substantially more visible in user-generated language than purchase-related orientations. This pattern closely parallels the findings observed for trust-related discourse and further supports the distinction between emotional expression and behavioral discourse within AI-saturated digital environments.

### 4.11 Category-level purchase-related discourse

To further examine RQ2, purchase-related discourse was analyzed across AI perception categories, and the corresponding findings are reported in **Table 9**. Consistent with the aggregate findings, purchase-related discourse remained highly infrequent across all categories. In every AI perception category, the overwhelming majority of comments contained no explicit purchase-related expression.

**Table 9:** Category-level purchase-related discourse.

AI perception type	Negative_N	Negative_%	Neutral_N	Neutral_%	Positive_N	Positive_%	Total_N
Lack_Of_Authenticity	310	0.13	245,747	99.46	1,020	0.41	247,077
Scriptedness	270	0.06	480,049	99.34	2,940	0.61	483,259
Automation_Perception	335	0.05	678,696	99.63	2,180	0.32	681,211
Perceived_Artificiality	215	0.07	305,258	99.73	620	0.20	306,093
Robotic_Communication_Style	165	0.08	197,984	99.83	180	0.09	198,329
Uncanny_Valley_Perception	155	0.04	366,703	99.90	227	0.06	367,085
Chi-square	2,815.95						
p-value	<.001						
df	10						
Effect size (Cramér’s V):	.03						

Although modest category-level variation was observed, purchase-related discourse appeared somewhat more frequently in categories such as scriptedness, lack of authenticity, and automation perception. Nevertheless, the observed frequencies remained extremely low across all categories.

Although category-level differences reached statistical significance (χ² = 2,815.95, df = 10, p <.001, Cramér’s V = .03), the overall practical magnitude of the association remained small. Negative purchase-related discourse was negligible across all AI perception types.

These findings indicate that AI perception categories differ far more clearly in emotional expression than in purchase-related discourse. While users readily articulate affective reactions to perceived AI-generated or AI-assisted content, they rarely express explicit purchase orientations within naturally occurring comment environments.

Overall, the category-level findings further reinforce the interpretation that emotional appraisal constitutes the dominant form of response in this dataset, whereas purchase-related discourse remains sparse, weakly differentiated, and discursively peripheral. Together with the trust-related discourse results, these findings suggest that explicit behavioral discourse occupies a comparatively limited role within AI-related user expression.

### 4.12 Relative strength of emotional expression and behavioral discourse indicators

H3 proposed that AI perception would demonstrate a stronger and more consistent association with negative emotional expression than with trust-related and purchase-related behavioral discourse indicators. The findings across Sections 4.6 to 4.11 support this hypothesis.

The contrast across outcome domains is substantial, as shown in **Table 10**. AI perception was associated with a 37.24 percentage-point increase in negative emotional expression, with negative sentiment appearing in 67.12% of AI-perception comments compared with 29.88% of comments without AI perception. By contrast, the difference in trust-related discourse between AI-perception and non-AI-perception comments was only 0.21 percentage points. Purchase-related discourse remained extremely rare throughout the corpus, appearing in only 0.37% of AI-perception comments and remaining absent in 99.63% of them.

**Table 10:** Relative strength of emotional expression and behavioral discourse indicators.

Outcome indicator	Main contrast	Interpretation
Negative emotional expression	67.12% vs 29.88%; +37.24 pp	strong expressive difference
Trust-related discourse	0.74% vs 0.53%; +0.21 pp	sparse and weak
Purchase-related discourse	0.37% total in AI-perception comments	highly sparse
Overall pattern	emotional expression >> behavioral discourse	supports expressive decoupling

Accordingly, emotional expression was not only considerably more prevalent but also much more clearly differentiated by AI perception than either trust-related or purchase-related discourse indicators. This contrast was evident both in the aggregate analyses and in the category-level results, where emotional expression displayed substantial variation across AI perception types while behavioral discourse indicators remained sparse and only weakly differentiated.

This overall pattern supports the concept of expressive decoupling. The findings indicate that AI perception is strongly reflected in emotional and affective language, whereas explicit behavioral discourse remains weak, infrequent, and contextually constrained.

Importantly, these findings should not be interpreted as evidence that AI perception lacks behavioral relevance. The dataset does not capture observable actions or real-world behavioral outcomes. Rather, the results suggest that within this AI-saturated YouTube comment environment, emotional appraisal becomes substantially more visible in discourse than explicit trust-related or purchase-related orientation.

### 4.13 Descriptive temporal patterns in AI-related emotional expression

RQ3 examined how AI-related emotional expressions and comment volumes vary descriptively over time. The temporal analysis was conducted descriptively and does not support causal interpretation. The analysis does not control for contextual factors such as platform policy changes, algorithmic modifications, shifts in video content, or broader technological and societal developments. Accordingly, the findings should be interpreted as temporal patterns within the observed corpus rather than as evidence of historical causal effects.

The temporal sentiment analysis indicated that negative emotional expression remained consistently prominent throughout the observation period, although its intensity and distribution varied across time and AI perception categories. In earlier periods, some AI perception categories displayed relatively more balanced distributions between positive and negative emotional expression. In later periods, however, negative emotional expression became increasingly pronounced across several categories, particularly robotic communication style, lack of authenticity, automation perception, and uncanny valley perception.

Scriptedness demonstrated a comparatively distinct temporal profile. Across multiple periods, scriptedness-related comments showed relatively higher levels of positive emotional expression than other AI perception categories. This pattern is consistent with the category-level sentiment findings reported in Section 4.7, where scriptedness was the only AI perception category associated with a predominantly positive emotional profile.

The temporal findings therefore further support the interpretation that different forms of AI perception are associated with meaningfully different affective patterns in user-generated discourse. Overall, the descriptive temporal analysis indicates that AI-related emotional expression is not static but varies across time and perception type within AI-saturated digital environments.

Nevertheless, these temporal patterns should be interpreted as descriptive fluctuations in discourse rather than as evidence of direct technological, societal, or behavioral causation.

### 4.14 Descriptive temporal patterns in comment volume

The second component of RQ3 concerned temporal variation in comment volume. The descriptive volume analysis indicated that AI-related comment activity was unevenly distributed across the observation period. Rather than remaining stable over time, the volume of AI-related comments fluctuated across different periods, with some intervals characterized by substantially higher levels of discourse activity. These patterns suggest that AI-related communication became more visible and discursively prominent during particular phases of the dataset.

At the same time, the temporal distribution of comment volume should be interpreted cautiously. Variations in comment activity may reflect numerous overlapping influences, including platform growth, shifts in recommendation systems, changes in content availability, fluctuations in public attention toward AI technologies, evolving search strategies, and broader transformations in digital communication environments. Because the analysis does not isolate or control for these contextual dynamics, the findings should not be interpreted as evidence that AI visibility directly caused changes in comment volume.

Taken together, the descriptive temporal analyses indicate that both emotional expression and AI-related discourse activity vary across time within the observed corpus. These patterns provide contextual insight into the evolving visibility and emotional articulation of AI-related discourse in large-scale digital environments while remaining strictly descriptive in interpretive scope.

### 4.15 Robustness and low base-rate interpretation

The findings were further evaluated with attention to robustness, class imbalance, and low base-rate interpretation. Across the analyses, the central pattern remained stable: AI perception was consistently more strongly reflected in negative emotional expression than in trust-related or purchase-related discourse.

Category-level analyses further indicated that this pattern was not driven by a single dominant AI perception category. Instead, elevated levels of negative emotional expression were observed across multiple categories, particularly robotic communication style, lack of authenticity, automation perception, and uncanny valley perception.

At the same time, the sparse distribution of trust-related and purchase-related discourse requires careful interpretation. Trust-related discourse appeared in only 0.74% of comments, while purchase-related discourse appeared in 0.37%. Given these very low frequencies, these indicators are more appropriately interpreted as limited discursive signals rather than stable or comprehensive representations of behavioral orientation.

Taken together, the overall evidence supports the conceptual interpretation of expressive decoupling. AI perception is associated with substantial variation in emotional expression, whereas explicit behavioral discourse remains comparatively rare, weakly differentiated, and discursively peripheral.

Within the context of this AI-saturated digital environment, the findings are best understood as reflecting a distinction between the visibility of affective expression and the comparatively limited articulation of behavioral orientation in user-generated discourse.

## 5 Discussion

### 5.1 Overview of findings

This study set out to examine how the perceived presence of artificial intelligence is associated with emotional expression and behavioral discourse in large-scale, naturalistic digital environments. Drawing on more than 2.3 million user-generated comments, the analysis reveals a clear and consistent pattern: AI perception is strongly associated with negative emotional expression, while its association with behavioral discourse indicators—specifically trust-related and purchase-related expressions—remains weak, rare, and contextually constrained.

This pattern emerges across multiple dimensions of the analysis. Comments containing AI perception exhibit substantially higher levels of negative emotional expression than comments without AI perception. Emotional expression also varies meaningfully across AI perception categories, indicating that AI perception is not a monolithic construct but a multidimensional appraisal field characterized by distinct emotional profiles. In contrast, trust-related and purchase-related discourse appear only rarely throughout the corpus and display relatively limited variation across AI perception conditions.

The temporal analyses further suggest that emotional expression varies across time and AI perception categories. However, these temporal patterns remain descriptive and should not be interpreted as evidence of causal effects.

Taken together, the findings support the central proposition of the study: within AI-saturated digital environments, emotional responses to perceived AI are substantially more visible in user-generated discourse than explicit behavioral discourse. This pattern is conceptualized as expressive decoupling, reflecting a divergence between affective expression and its discursive behavioral articulation (16, 17).

### 5.2 AI perception and negative emotional expression

The findings provide strong support for H1, demonstrating that AI perception is associated with substantially higher levels of negative emotional expression in user-generated comments. The magnitude of the observed difference suggests that this relationship is not merely statistically significant but also meaningful from a substantive perspective.

From a psychological perspective, this result aligns with existing research on algorithm aversion, perceived artificiality, and the uncanny valley (7–9), which suggests that individuals may experience discomfort, skepticism, or reduced emotional resonance when interacting with AI-mediated content. However, the present study extends this literature by demonstrating that such responses are not limited to controlled experimental settings or self-reported attitudes. Instead, they are actively expressed in large-scale, naturalistic digital discourse.

This finding is particularly relevant for digital mental health research. Emotional expression in online environments can be understood as an externalization of cognitive–affective appraisal processes (18, 20). The consistent presence of negative emotional language in AI-related comments suggests that AI visibility may function as a salient interpretive cue associated with how users linguistically express emotional reactions to digital content.

At the same time, it is important to emphasize that the study captures expressed emotional language rather than internal psychological states or clinical outcomes. Consequently, the findings should be interpreted as evidence of discursive emotional expression in AI-related environments rather than as evidence of psychiatric symptoms, psychological impairment, or broader mental health consequences.

### 5.3 Heterogeneity in AI perception and emotional response

The results also provide clear support for H2, showing that emotional expression varies significantly across different AI perception types. This finding challenges the dominant tendency in prior research to treat AI perception as a single, uniform construct (6, 13). In particular, categories such as robotic communication style and lack of authenticity are associated with the highest levels of negative emotional expression, suggesting that perceived violations of humanness, sincerity, or relational presence are especially salient in shaping user reactions.

By contrast, scriptedness displays a markedly different pattern, with a higher proportion of positive expressions. This suggests that not all AI-related cues are interpreted as aversive; some may be perceived as structured, predictable, or even functionally desirable in certain contexts. The comparatively positive profile of scriptedness further highlights the importance of distinguishing among different forms of perceived AI involvement rather than treating AI perception as a single evaluative construct.

This heterogeneity has important theoretical implications. It indicates that AI perception operates as a multidimensional appraisal field (13, 14), in which different perceptual cues activate distinct interpretive framings within user discourse. As a result, emotional responses to AI cannot be adequately understood through aggregated or monolithic measures. Instead, a more fine-grained approach is required to capture how users differentiate among forms of perceived artificiality, automation, and authenticity in real-world digital environments.

### 5.4 AI perception and trust-related discourse

The findings related to RQ1 indicate that AI perception shows only a weak and practically limited association with trust-related discourse. Although slight differences are observed, trust-related expressions remain extremely rare across the dataset.

This result should be interpreted with caution. The absence of frequent trust-related discourse does not imply that trust is irrelevant in human–AI interaction. Rather, it suggests that explicit expressions of trust or distrust are not a dominant feature of user-generated comments in this context. Trust may operate implicitly, may be embedded within other forms of expression, or may simply remain unarticulated in short-form digital interactions.

From a methodological perspective, this finding reinforces the importance of distinguishing between latent psychological constructs and their observable linguistic manifestations (17, 18). The study does not measure trust as a psychological state; it captures only explicit trust-related language. Consequently, the findings should not be interpreted as evidence of stable trust formation, trust erosion, or broader relationship dynamics between users and AI systems.

Instead, the results indicate that trust-related discourse occupies a relatively limited position within AI-related user expression, even in a highly AI-saturated communicative environment.

### 5.5 AI perception and purchase-related discourse

The findings related to RQ2 indicate that purchase-related discourse is extremely rare and only weakly associated with AI perception. This pattern is consistent with the overall descriptive structure of the dataset, in which explicit purchase-related expressions appear in less than one percent of comments.

These results highlight the distinction between discursive expression and observable behavior in digitally mediated environments (16, 17). Within this context, user-generated comments appear to function primarily as spaces for emotional and evaluative expression rather than as direct indicators of purchasing orientation or observable action (40). Although users frequently articulate affective reactions to AI-related content, explicit purchase-related language remains largely absent.

This finding carries important methodological and theoretical implications. It suggests that emotional responses to AI do not necessarily become visible through purchase-related discourse in low-commitment digital environments such as YouTube comment sections. The limited presence of purchase-related expressions should not be interpreted as evidence that AI perception lacks behavioral relevance. Rather, it indicates that explicit behavioral orientation is only minimally articulated within this form of naturally occurring online discourse.

The relative absence of purchase-related discourse is nevertheless theoretically informative because it highlights the limited extent to which emotional reactions become explicitly translated into behavioral language within user-generated discourse. In this respect, the findings parallel those observed for trust-related discourse and further reinforce the distinction between affective expression and behavioral discourse in AI-saturated digital environments.

### 5.6 Expressive decoupling between emotion and behavioral discourse

A central contribution of the study lies in the support for H3, demonstrating that AI perception is considerably more strongly associated with emotional expression than with behavioral discourse indicators. This contrast is substantial: emotional expression displays clear and meaningful variation across AI perception conditions, whereas trust-related and purchase-related discourse remain infrequent and only weakly differentiated.

This pattern is consistent with the concept of expressive decoupling, referring to a divergence between affective responses and their explicit discursive articulation in relation to trust or purchase orientation (16, 17, 23). In other words, users may linguistically articulate strong emotional reactions to perceived AI without simultaneously verbalizing corresponding behavioral orientations within their comments (15).

This finding should be interpreted within the constraints of the dataset and the communicative environment under examination. The analysis does not assess observable behavior and therefore does not evaluate whether emotional responses ultimately influence decision-making or real-world action. Within this perspective, the study captures an early interpretive and discursive layer of AI-related response rather than downstream behavioral outcomes such as purchasing, engagement, recommendation uptake, or well-being.

Instead, the findings indicate that within this AI-saturated digital context, emotional expression is substantially more visible and more frequently articulated than explicit behavioral discourse. This distinction is important for interpreting large-scale user-generated text, where emotional appraisal may become highly salient in language even when behavioral orientation remains implicit or unexpressed. More broadly, the findings suggest that emotional expression and behavioral discourse should not be assumed to operate at comparable levels of visibility within digital communication environments, particularly when user responses are examined through naturally occurring textual data.

### 5.7 Descriptive temporal patterns

The results related to RQ3 indicate that both emotional expression and comment volume vary over time. However, these patterns are strictly descriptive and do not support causal interpretation. Temporal variation may reflect multiple factors, including platform dynamics, changes in content, evolving public awareness of AI, and broader technological developments. Because the analysis does not control for these factors, it cannot isolate the specific drivers of observed temporal changes.

Nevertheless, the temporal patterns provide useful contextual insight. They suggest that AI-related discourse is not static and that the visibility and emotional articulation of AI-related expression may vary across different periods within digital communication environments.

These findings are broadly consistent with literature documenting temporal variation in large-scale digital discourse (30). However, the present results should be interpreted as exploratory descriptive patterns rather than evidence of technological adaptation, normalization, or other longitudinal processes.

### 5.8 Implications for digital mental health and human–AI interaction

This study contributes to digital mental health research by demonstrating that large-scale user-generated discourse can provide valuable insight into how users linguistically express emotional reactions to AI-related content in real-world environments. The findings suggest that AI visibility is associated with increased negative emotional expression, highlighting the importance of considering affective responses in the design and deployment of AI-mediated systems. In this respect, the study contributes to emerging discussions in digital psychiatry and digital mental health concerning how technologically mediated environments shape large-scale emotional expression and psychologically meaningful communication patterns (35).

At the same time, the study emphasizes the need for conceptual precision. Emotional expression in digital discourse should not be equated with clinical outcomes or behavioral change (17, 18). Instead, it represents one layer of psychological response that interacts with, but does not fully determine, other forms of cognition and behavior.

For human–AI interaction research, the findings underscore the importance of distinguishing between different types of AI perception and their corresponding patterns of emotional and discursive expression (41). Not all AI-related cues produce the same responses, and understanding this variation is essential for designing systems that are perceived as authentic, trustworthy, and psychologically acceptable.

The study also contributes to the emerging consumer-AI literature by examining how users publicly articulate perceptions of artificiality, automation, authenticity, and emotional discomfort within naturally occurring digital environments. Because the analysis focuses on discursive expression rather than downstream outcomes, the findings should be interpreted as complementary to research examining purchasing behavior, engagement, recommendation uptake, trust formation, well-being, or other behavioral and psychological consequences of AI use.

Taken together, the findings provide ecological insight into how AI-related perceptions become socially expressed within large-scale user-generated communication environments.

### 5.9 Limitations and future research

Several limitations should be acknowledged when interpreting the findings of this study. The dataset consists of observational user-generated comments collected from YouTube, a platform characterized by highly performative and affectively expressive communication dynamics. Social media comment environments often amplify emotional reactions, sarcasm, identity signaling, exaggeration, and polarized discourse. Consequently, the observed emotional expressions and AI-perception patterns may reflect heightened discursive performance rather than stable internal attitudes or offline behavioral tendencies. This issue is further complicated by the nature of large-scale social media discourse, where meaning is often context-dependent, linguistically fluid, and shaped by informal communication styles. Although the classification pipeline demonstrated acceptable validation performance, LLM-assisted and transformer-based models may still misclassify sarcasm, irony, slang, ambiguous phrasing, or culturally specific expressions, which remains an important methodological limitation when interpreting emotionally charged online discourse.

The analysis relies on textual indicators, which capture expressed discourse rather than internal psychological states or actual behavior (18, 20). The study does not measure actual purchasing behavior, engagement behavior, trust formation, recommendation uptake, well-being, psychiatric outcomes, or long-term attitudinal change. Variables such as purchase-intention proxy and trust-related discourse should therefore be interpreted as discourse-level linguistic indicators rather than direct behavioral measurements.

The dataset represents a highly AI-saturated communication environment. Because the retrieval strategy intentionally targeted AI-related communication cues, influencer authenticity concerns, sponsorship discourse, and perceptions of artificiality, AI perception was highly prevalent throughout the cleaned corpus. The findings should therefore be interpreted as reflecting AI-salient digital discourse rather than generalized online communication environments. Future studies should examine whether similar expressive patterns emerge in contexts where AI-related cues are less visible or less structurally salient.

Although transformer-based classifiers and LLM-assisted weak supervision provide substantial scalability advantages for large-scale discourse analysis, automated classification systems inevitably involve uncertainty. Consequently, the resulting labels should be interpreted as probabilistic discourse indicators rather than definitive psychological assessments.

Although the analyzed comments were publicly accessible, the use of public digital traces does not eliminate all ethical considerations. Large-scale computational analysis of user-generated discourse may still raise concerns related to contextual privacy, platform expectations, and ethical interpretation of online expression. To minimize such risks, the study focused exclusively on aggregate discourse-level patterns and avoided individual-level profiling or identifiable user analysis.

The present study captures an early interpretive and discursive layer of AI-related audience response rather than downstream behavioral outcomes. In this sense, the study is best understood as complementary to consumer-AI research examining measurable outcomes such as purchasing behavior, engagement metrics, recommendation adoption, or subjective well-being.

Future research should combine discourse analysis with experimental, longitudinal, behavioral, and multimodal approaches to better understand how AI-related emotional expression relates to decision-making processes and real-world behavioral outcomes.

Finally, the study was not preregistered. Although the analytical procedures, classification strategy, and interpretive boundaries were explicitly documented and made publicly available through an open repository, the absence of preregistration limits the extent to which analytical decisions can be evaluated independently of the observed data. Future research may benefit from preregistered designs and fully open computational workflows to further strengthen transparency and reproducibility in large-scale AI discourse research (36).

## 6 Conclusion

This study examined how the perceived presence of artificial intelligence is reflected in emotional expression and behavioral discourse within large-scale, naturalistic digital environments. Drawing on a dataset of more than 2.3 million user-generated comments, the findings reveal a consistent and robust pattern: AI perception is strongly associated with negative emotional expression, yet shows only limited and contextually constrained associations with explicit trust-related and purchase-related discourse.

By adopting a discursive and observational approach, the study moves beyond intention-based and experimental research designs and provides insight into how psychological responses to AI are articulated in real-world communication environments. The findings demonstrate that emotional reactions to perceived AI are both frequent and heterogeneous, varying significantly across different AI perception types. In contrast, behavioral discourse indicators remain rare and only weakly differentiated, highlighting the importance of distinguishing between affective expression and expressed behavioral orientation.

A central contribution of the study is the identification of a pattern consistent with expressive decoupling, which helps explain the divergence between emotional expression and behavioral discourse in AI-saturated digital contexts. Rather than assuming a direct translation from affective response to behavioral intention, the results suggest that users may articulate strong emotional reactions to AI without simultaneously expressing corresponding trust-related or purchase-related orientations (16, 17). This distinction is particularly important for interpreting large-scale textual data, where linguistic expression does not necessarily reflect internal psychological states or observable behavior.

These findings contribute to digital mental health research by demonstrating how AI-related perceptions are externalized through language in naturally occurring environments. The results indicate that AI visibility functions as a salient interpretive cue associated with patterns of emotional expression, while also underscoring the need for caution when interpreting textual indicators as evidence of behavioral change or clinical outcomes. At the same time, the study contributes to human–AI interaction research by highlighting the multidimensional nature of AI perception and the importance of differentiating between distinct perceptual cues and their corresponding emotional effects.

Several limitations should be acknowledged. The analysis relies on observational data from a single platform and on textual indicators rather than direct measures of psychological states or behavior. In addition, the low base rate of behavioral discourse variables constrains inferential interpretation. Accordingly, the findings should be understood as patterns of expressed discourse within an AI-saturated environment rather than as evidence of causal relationships or behavioral outcomes.

Future research should extend this line of inquiry by integrating behavioral data, experimental methods, and longitudinal designs to better understand how emotional responses to AI relate to decision-making processes over time. Multimodal approaches combining textual, behavioral, and physiological data may further enrich the understanding of human–AI interaction in complex digital ecosystems.

In conclusion, this study demonstrates that in contemporary digital environments characterized by high AI visibility, emotional responses are prominently expressed, while behavioral discourse remains limited and context-dependent. Recognizing this distinction is essential for advancing theoretical and empirical understanding of digital mental health and human–AI interaction, particularly as researchers increasingly rely on large-scale user-generated data to examine psychological expression in digital environments.

## Data Availability

The datasets, analytical code, annotation resources, prompts, and **Supplementary Materials** supporting the findings of this study are publicly available in the Zenodo repository: https://doi.org/10.5281/zenodo.20424036. The repository contains the materials necessary to support transparency, reproducibility, and methodological verification of the study.
