# Supplementary material for: Psychological decoupling in responses to AI: emotional reactivity and behavioral discourse in digital environments
Source: Front Psychiatry. 2026 Jun 24;17:1839044. doi: 10.3389/fpsyt.2026.1839044 (PMC13343222; doi:10.3389/fpsyt.2026.1839044)
Supplement: Supplementary file 1 [file DataSheet1.pdf]

Supplementary Material

This supplementary appendix provides detailed methodological documentation and reproducibility materials supporting the analyses reported in the main manuscript.

Supplementary Methodological Appendix for: Psychological Decoupling in Responses to AI: Emotional Reactivity and Behavioral Discourse in Digital Environments

Appendix A. YouTube Search Criteria

The YouTube dataset was retrieved using 47 search criteria designed to capture discourse related to sponsorship disclosure, influencer marketing, authenticity, perceived artificiality, AI-related communication, automation cues, and audience evaluations of digital content.

1. sponsored review
2. paid partnership review
3. influencer advertisement
4. sponsored content review
5. brand collaboration review
6. this video is sponsored
7. advertisement disclosure influencer
8. influencer promotion review
9. honest review influencer
10. fake influencer review
11. influencer authenticity
12. influencer honesty
13. misleading influencer marketing
14. deceptive online marketing
15. influencer exposed
16. can I trust this influencer
17. is this review genuine
18. review real or fake
19. authenticity in social media
20. manipulative influencer content
21. AI generated content review
22. AI influencer review
23. artificial influencer
24. robotic communication style
25. automated content creator
26. scripted online content
27. AI voice review
28. synthetic media review
29. fake human AI
30. uncanny AI content
31. machine generated content
32. AI generated videos
33. virtual influencer review
34. AI communication style
35. wellness influencer review
36. lifestyle influencer review
37. fitness influencer review
38. daily routine influencer
39. aesthetic lifestyle content
40. morning routine review
41. wellness product review

42. supplement review influencer
43. skincare influencer review
44. product recommendation influencer
45. audience reaction influencer
46. social media trust issues
47. influencer credibility review

Appendix B. Data Collection Procedure

Data were collected from YouTube using the official YouTube Data API v3. The collection pipeline queried YouTube through the search, videos, and commentThreads endpoints. First, videos were retrieved using the predefined search criteria listed in Appendix A. The search request was restricted to video results and used the snippet parameter with a maximum of 50 results per request. Second, video-level metadata were retrieved through the videos endpoint using the snippet and statistics parameters. Third, top-level comments were collected through the commentThreads endpoint using plain-text comment formatting. Data extraction was conducted on videos published between January 2010 and January 2025. For each video, the collection procedure stored video-level metadata including video identifier, title, description, channel title, publication date, view count, like count, comment count, and the search query through which the video was retrieved. At the comment level, the stored fields included comment identifier, video identifier, author display name, comment text, publication date, comment like count, and total reply count. The unit of analysis in the study is the individual comment. The collection script implemented pagination for both video search results and comment retrieval. Comment retrieval was conducted with the maximum API-supported page size of 100 comments per request. Videos were eligible for comment extraction when they had at least one view and at least one comment. The pipeline used SQLite for structured data storage and maintained a progress table to allow interrupted collection processes to resume from the last completed query, page token, and API-key index. To improve collection stability, the script included retry handling, request backoff, request-level logging, response caching, batch processing of video metadata, and API quota management. When quota-related errors were encountered, the script rotated to the next available API credential. These procedures were implemented to reduce data loss during long-running collection and to ensure that collection could be resumed consistently after interruption. English-language filtering was applied at the video level using the langdetect library. The language detector seed was fixed to improve reproducibility. Videos were retained when the combined title and description were detected as English. Videos with non-English title-description combinations were excluded before comment extraction. This procedure was used to increase the likelihood that the resulting comment corpus reflected English-language discourse while maintaining a scalable filtering strategy suitable for large-scale YouTube data collection.

Appendix C. Inclusion and Exclusion Rules

The raw comment corpus was cleaned using a multi-stage preprocessing pipeline. First, comments with missing text values were removed. All remaining comments were converted into string format and processed at the comment-text level. Only English-language comments were retained. Language filtering was performed using the langdetect library, and comments were included only when the detected language was English. Text normalization was then applied to produce a cleaned comment field. The normalization procedure converted text to lowercase, removed emojis, removed URLs, removed punctuation marks, removed numerical characters, collapsed multiple spaces into a single space, and trimmed leading and trailing whitespace. This procedure was designed to reduce non-linguistic noise while preserving semantically meaningful AI-perception cues. Low-information and noise-like comments were excluded using an operational rule based on comment length and boilerplate content. Comments containing fewer than three words were removed unless they contained theoretically relevant signal terms, including “ai,” “fake,” “bot,” “scripted,” “robotic,” or “creepy.” This exception was applied to avoid removing short but substantively relevant expressions of AI perception. In addition, common low-information boilerplate comments, such as “nice video,” “great video,” “thanks for sharing,” “love this,” “awesome,” and “first comment,” were excluded. Duplicate removal was

conducted after text normalization. Exact duplicate comments were identified based on the cleaned text field and removed. This approach ensured that repeated comments with identical normalized content were not overrepresented in the analytical corpus. After cleaning, each retained comment was assigned a unique global comment identifier.

Appendix D. AI-Perception Taxonomy and Illustrative Examples

The AI-perception taxonomy was developed to capture different forms of perceived artificiality, automation, and non-human communication style in user-generated discourse. Each category was operationalized through semantic interpretation rather than simple keyword matching alone. Table D1 presents illustrative examples of positive and negative cases used during category development and manual validation.

AI-Perception Category	Operational Description	Illustrative Positive Example	Illustrative Negative Example
**Automation Perception**	References to algorithmic, automated, or machine-driven production	"This feels completely automated."	"This creator explains things naturally."
**Robotic Communication Style**	Perception that communication sounds mechanical, emotionless, or machine-like	"The voice sounds robotic and unnatural."	"The delivery feels human and conversational."
**Scriptedness**	References to repetitive, formulaic, overly structured, or pre-planned communication	"This sounds heavily scripted."	"The conversation feels spontaneous."
**Lack of Authenticity**	Expressions suggesting insincerity, emotional artificiality, or deceptive presentation	"None of this feels genuine."	"This review feels authentic and honest."
**Perceived Artificiality**	Explicit recognition of artificial, synthetic, or AI-like qualities	"This content feels artificially generated."	"This feels like a real personal opinion."
**Uncanny Valley Perception**	Expressions of discomfort arising from near-human but not fully human presentation	"This is creepy in a strangely human way."	"Nothing about this feels unsettling."

Boundary and Overlap Considerations

Some comments contained overlapping AI-perception cues. For example, comments describing communication as both "robotic" and "fake" could simultaneously reflect robotic communication style and lack of authenticity. Similarly, comments referring to "AI-generated fake humans" could overlap between perceived artificiality and uncanny valley perception. Because naturally occurring discourse often combines multiple evaluative dimensions, category boundaries were treated as semantically related rather than mutually exclusive psychological states.

Classification Principle

The classification process prioritized contextual semantic meaning over isolated keyword occurrence. For example, the term "scripted" was not automatically classified as scriptedness unless the surrounding context referred to formulaic or artificial communication. Similarly, references to "fake" were interpreted differently depending on whether the comment referred to deceptive presentation, synthetic media, or unrelated colloquial usage.

Appendix E. Weak Supervision and LLM-Assisted Pre-Labeling

The initial AI-perception labels were generated through an LLM-assisted weak supervision procedure using the locally deployed Ollama gemma3:4b model. The model was used to assign preliminary category labels to selected comments before supervised transformer-based classification. These labels were not treated as final ground truth, but as weak supervision signals for scalable model development. For each comment, the model was instructed to classify the text into the single most relevant AI-perception category or to assign "None" when no category was applicable. The six target categories were: perceived artificiality, scriptedness, robotic communication style, uncanny valley perception, automation perception, and lack of authenticity. Although the output file stored the results in one-hot column format, each comment received only one primary LLM-generated category during this pre-labeling stage. The prompt used a definition-based classification structure. The model was first instructed to act as an expert in social media comment analysis. It was then provided with the six AI-perception categories and a "None" option, followed by concise operational definitions for each category. The model was explicitly instructed to select the single most relevant category, avoid explanatory reasoning, and return exactly one category name.

A simplified version of the prompt structure was as follows:

```
PROMPT_TEMPLATE = """
You are an expert in analyzing social media comments.

Task:
Classify the following comment into the single most relevant category:
1. Perceived Artificiality
2. Scriptedness
3. Robotic Communication Style
4. Uncanny Valley Perception
5. Automation Perception
6. Lack of Authenticity
7. None

Definitions:
- Perceived Artificiality: The comment suggests the content feels
fake, unnatural, or artificial.
- Scriptedness: The comment implies the message feels rehearsed or
pre-written.
- Robotic Communication Style: The comment describes mechanical or
robotic language.
- Uncanny Valley Perception: The comment expresses discomfort due to
almost-human behavior.
- Automation Perception: The comment suggests the content is generated
by AI or algorithms.
- Lack of Authenticity: The comment questions sincerity or
genuineness.

Rules:
- Choose the SINGLE MOST RELEVANT category.
- If none apply, respond with "None".
- Do not explain your reasoning.
- Respond with exactly one category name.

Comment:
"{comment}"
"""
```

The LLM response was parsed into category flags. When the response matched one of the six predefined categories, the corresponding category column was assigned a value of 1 and all other category columns were assigned 0. When the response was “None” or could not be parsed, all category columns were assigned 0. A short delay was included between local model calls to maintain stable processing. Because LLM-generated labels may contain noise, ambiguity, and category-boundary errors, these pre-labels were subsequently evaluated through manual validation and used as weak supervision rather than as definitive human-coded labels.

Appendix F. Manual Validation Procedure

To evaluate the quality of the LLM-assisted weak supervision labels, a structured manual validation procedure was conducted. Validation was performed independently by two human reviewers: the author and a faculty supervisor with expertise in digital communication and computational text analysis. For each AI-perception category, a random sample consisting of 250 positive and 250 negative instances was selected from the pre-labeled dataset. The sampled comments were manually reviewed using the operational category definitions described in Appendix D. The purpose of this stage was to assess the degree of alignment between LLM-generated labels and human semantic interpretation. The reviewers independently evaluated whether the assigned category appropriately reflected the semantic meaning of the comment. Cases involving ambiguity, overlapping category cues, sarcasm, or unclear contextual interpretation were subsequently discussed and resolved through consensus-based adjudication. Inter-rater agreement was evaluated using Cohen’s kappa coefficient to assess labeling consistency beyond chance agreement. The analysis yielded an approximate Cohen’s kappa value of $\kappa = .76$, indicating substantial agreement between reviewers. This level of agreement supported the reliability of the operational category definitions and the use of the weak supervision framework for large-scale pre-labeling. Following manual validation, the validated weak labels were used in the subsequent supervised transformer-based classification stage.

Appendix G. Transformer Training and Classification Thresholds

Following the weak supervision stage, supervised transformer-based classification models were trained separately for each AI-perception category. The classification architecture used the RoBERTa-base transformer model implemented through the Hugging Face Transformers framework. Independent binary classifiers were trained for the following categories: automation perception, scriptedness, robotic communication style, perceived artificiality, uncanny valley perception, and lack of authenticity.

Training Procedure

For each category, the dataset was divided into training, validation, and test subsets using stratified sampling. First, 20% of the data were reserved as the test set. The remaining data were then split into training and validation subsets, with validation sampling conducted stratified by label distribution. Random seed consistency was maintained using random_state=42.

Tokenization was performed using the RoBERTa tokenizer with:

- maximum sequence length = 128,
- truncation enabled,
- max-length padding.

The models were trained for four epochs using:

- batch size = 16,
- learning rate = 2×10^{-5},
- weighted cross-entropy loss,
- label smoothing = 0.1.

To reduce class imbalance effects, balanced class weights were computed from the training distribution and incorporated into the loss function. Early stopping was implemented with a patience parameter of two evaluation cycles. Model selection was based on validation F1 score. Training and inference were conducted using PyTorch and the Hugging Face Trainer framework. GPU acceleration was enabled when CUDA-compatible hardware was available.

Evaluation Metrics

Model performance was evaluated using:

- accuracy,
- precision,
- recall,
- F1 score,
- confusion matrices.

Predictions on the test set were generated through argmax selection over the model logits.

Final Classification Logic

Following category-specific model training, all cleaned comments were processed through the six trained binary classifiers. For each comment, the models generated category-specific probabilities using softmax outputs. The probability associated with the positive class (p(class=1)) was extracted for each category.

The final category assignment procedure used a confidence threshold of 0.50. For each comment:

1. The category with the highest predicted probability was identified.
2. If the highest probability was greater than or equal to 0.50, the corresponding category was assigned as the final label.
3. If all category probabilities were below 0.50, the comment was assigned a “None” category.

This procedure was used to reduce low-confidence classifications and to avoid forced assignment of weakly related comments into AI-perception categories.

Appendix H. Sentiment Analysis Procedure

Sentiment analysis was conducted using the Hugging Face Transformers sentiment-analysis pipeline with the pretrained model distilbert-base-uncased-finetuned-sst-2-english. This model is based on DistilBERT and was originally fine-tuned on the Stanford Sentiment Treebank v2 (SST-2) dataset for binary English-language sentiment classification. All cleaned comments were processed in batches of 32 comments during inference. Text truncation was enabled to ensure compatibility with model input constraints. For each comment, the model returned a sentiment label and associated confidence score. The original model outputs were operationalized into discourse-level sentiment categories through direct label mapping:

- POSITIVE → Positive
- NEGATIVE → Negative

The pipeline did not use an additional probability threshold beyond the model’s native prediction procedure. Sentiment labels were assigned directly according to the highest-confidence output generated

by the pretrained classifier. The sentiment analysis stage was intended to capture broad affective orientation in user-generated discourse rather than clinically validated emotional states or psychological outcomes. Consequently, the resulting sentiment labels should be interpreted as indicators of expressed affective language within comments rather than direct measurements of internal emotional conditions.

Because transformer-based sentiment models may misinterpret sarcasm, irony, internet slang, ambiguous expressions, or culturally specific language patterns, the sentiment outputs were treated as probabilistic discourse indicators rather than exact representations of subjective emotional experience.

Appendix I. Trust and Purchase Discourse Detection

In addition to AI-perception and sentiment classification, the study included rule-based detection procedures for purchase-related discourse indicators. These procedures were designed to identify explicit self-reported purchase-oriented expressions within user-generated comments rather than actual purchasing behavior. Purchase-related discourse was operationalized through manually defined linguistic patterns implemented using regular expressions. Positive purchase-intention proxy patterns included expressions such as:

- “I bought”
- “I have bought”
- “I will buy”
- “I’ll buy”
- “I will try”
- “I’ll try”
- “I am buying”
- “ordered this”
- “going to try”

Negative purchase-orientation patterns included expressions such as:

- “not buying”
- “won’t buy”
- “would not buy”
- “would never buy”
- “skipping this”
- “no way I buy”
- “not going to buy”

Comments matching positive patterns were labeled as Positive, comments matching negative patterns were labeled as Negative, and all remaining comments were labeled as Neutral. The detection procedure prioritized explicit self-referential purchase language rather than implicit recommendation sentiment. This design choice was intended to reduce false-positive classifications arising from generalized product discussion, abstract evaluation, or metaphorical language use. The resulting variable was interpreted as a discourse-level purchase-intention proxy rather than a direct measure of actual consumer behavior, purchasing activity, or economic decision-making. Consequently, the findings should not be interpreted as evidence of real-world behavioral conversion or market outcomes. Because rule-based discourse detection may fail to capture sarcasm, indirect intention, contextual ambiguity, or culturally specific phrasing, the purchase-discourse indicators were treated as approximate linguistic signals rather than precise behavioral measurements.
